# Employment precariousness and the importance of the assessment of fatal occupational injuries on the Mortality Information System

**DOI:** 10.1590/S2237-96222023000300012.en

**Published:** 2023-10-30

**Authors:** Eliane de Freitas Drumond

**Affiliations:** 1 Instituto Mário Penna, Núcleo de Ensino e Pesquisa, Belo Horizonte, MG, Brazil

The research note entitled *Assessment of incompleteness of Mortality Information System records on deaths from external causes in the state of Rio Grande do Sul, 2000-2019* 1 presented results of an ecological time series study based on the analysis of deaths from external causes (ECs), specifically, from transport accidents, homicides, suicides and falls, occurred in Rio Grande do Sul, registered on the Mortality Information System (*Sistema de Informações sobre Mortalidade* - SIM), between 2000 and 2019. There were discrepancies in the decreasing trends observed for sociodemographic variables among the alarming number of deaths analyzed (n = 146,882), according to causes of death.

Fatal or non-fatal occupational injuries, although preventable, are a significant (and partially hidden) public health problem in Brazil. Underreporting of fatal occupational injuries (FOI) in the country, although not appropriately dimensioned, is recognized.^2^,^3^ FOI should be recorded in various information sources, including those from the Ministry of Health (SIM; Notifiable Health Conditions Information System – *Sistema de Informação de Agravos de Notificação* - SINAN; and, mainly, Hospital Information System of the Brazilian National Health System – *Sistema de Informações Hospitalares do Sistema Único de Saúde* - SIHSUS); from the Ministry of Labor (Annual Social Information List – *Relação Anual de Informações Sociais* - RAIS); and from the Social Security department (Occupational Accidents and Diseases Information System – *Sistema para Informação de Acidentes e Doenças do Trabalho* - SISCAT). Assessing underreporting in each of these sources poses a challenging due to the lack of a unifying variable, which would enable automatic data linkage between these information systems.^4^

A verbal autopsy study of deaths from ECs in Porto Alegre, state of Rio Grande do Sul, conducted in the early 1990s, showed an 80% underreporting of FOI among workers who died mainly from homicides and transport accidents.^5^ When comparing different sources of information on FOI in Palmas, state of Tocantins, Rodrigues and Santana found the lowest underreporting of FOI on the SIM (28.9%); falls accounted for 20% of FOI.^4^ In addition to the lowest underreporting of FOI observed by Rodrigues and Santana on the SIM, this system also offers the possibility of retrieving information on the deceased person’s occupation (variable 14), the completeness of which was not analyzed in the study by Barbosa et al.

Thus, the significance of the SIM as a source of information on FOI in the country stands out, given that this information system provides universal coverage and encompasses both formal and informal workers, regardless of the legal nature of the health facility (SUS or private) and the place of death.

Therefore, outstanding studies, such as that of Barbosa et al., could greatly contribute to a more accurate assessment of FOI in Brazil, by analyzing variable 49 in the death certificate (occupational injuries: yes/no/ignored), especially when there is accelerated employment precariousness (commonly referred to as uberization and platformization) in the country.
